# Immunohistochemical Expression of Platelet-Derived Growth Factor Receptors in Ovarian Cancer Patients with Long-Term Follow-Up

**DOI:** 10.1155/2012/851432

**Published:** 2012-09-23

**Authors:** Christine Vestergaard Madsen, Karina Dahl Steffensen, Marianne Waldstrøm, Anders Jakobsen

**Affiliations:** ^1^Department of Oncology, Vejle Hospital, Kabbeltoft 25, 7100 Vejle, Denmark; ^2^Institute for Regional Health Services Research, University of Southern Denmark, 5230 Odense, Denmark; ^3^Department of Pathology, Vejle Hospital, Kabbeltoft 25, 7100 Vejle, Denmark

## Abstract

*Introduction*. The well-documented role of the PDGF system in tumor growth and angiogenesis has prompted the development of new biological agents targeting the PDGF system. The aim of the present study was to analyze the expression of the PDGF-receptors in ovarian cancer and to investigate its relation to histopathological parameters and long-term overall survival. *Methods*. The immunohistochemical expression of PDGFR-**α** and PDGFR-**β** was investigated in tumor and stromal cells in 170 patients with histologically verified epithelial ovarian cancer. *Results*. Almost half of the tumor specimens showed high expression of PDGFR-**α** and PDGFR-**β** in tumor cells (43% and 41%) and in stromal compartments (32% and 44%). There was a significant association between high expression of PDGFR-**α** and high expression of PDGFR-**β** in both tumor and stromal cells. Coexpression of PDGFR-**α** and PDGFR-**β** in stromal cells was seen more often in serous adenocarcinomas than in nonserous adenocarcinomas. No clear correlation between PDGFR expression and longterm overall survival or clinical parameters was found. *Conclusions*. PDGFR-**α** and PDGFR-**β** were expressed in a subset of ovarian carcinomas but did not show significant prognostic importance in this material.

## 1. Introduction

Epithelial ovarian cancer is the most deadly gynecologic cancer in the Western world. The majority of patients are diagnosed in advanced stage which is a contributory factor to the poor prognosis of the disease. The current state- of-art in front-line treatment is aggressive surgical debulking followed by a combination of chemotherapy with platinum/taxane [1, 2]. Even though high response rates are seen, relapse often occurs within few years, and, in most cases, the therapy will then change from a curative to a palliative perspective. A higher degree of individualized treatment strategies based on validated prognostic or predictive markers may help improve the outcome and are therefore highly warranted in ovarian cancer. 

Results from recently published studies have shown that the addition of antivascular endothelial growth factor (VEGF) treatment to first-line chemotherapy may be beneficial for a fraction of ovarian cancer patients [3, 4], also in the treatment of the recurrent disease [5–7]. However, several other growth factors are involved in angiogenesis [8], among them the platelet-derived growth factor (PDGF) system. It plays a role in cell growth [9], chemotaxis [9, 10], pericytes recruitment, and stabilization of microvasculature [11, 12] as well as in the recruitment of fibroblast in tumor stroma [13, 14]. The PDGF system may also contribute to lymphatic metastases [15]. Furthermore, the system has been thought to be involved in the tumor evasion of the anti-VEGF treatment [16]. 

The PDGF isoforms (PDGF-AA, AB, BB, CC, DD) and receptors (PDGFR-*α*, PDGFR-*β*, *α*/*β*) are expressed by a variety of normal cells [9, 17]. Even though there does not seem to be a quite clear separation between the operating mechanisms of the receptors, PDGFR-*β* is known to affect the pericyte/endothelial cell interactions and pericyte formation [18, 19], whereas PDGFR-*α* is important for the fibroblastic cell/mesenchymal formation [18]. Signal transduction molecules are known to interact with both receptors [20].

Many malignant tumors are characterized by high expression of the ligands and/or the receptors [21–27] which has also been reported in ovarian cancer [28–35], and recent years have witnessed a rapid development of new targeted treatments against the PDGF pathway [36, 37]. However, so far we do not have generally accepted criteria for the selection of patients for the novel biological treatments, which accentuates the need for more knowledge about the PDGF system in ovarian cancer and also in its different histological subtypes. Furthermore, the utility of PDGFR as a possible prognostic or predictive biomarker has not been fully elucidated. 

Because of the evidence of the PDGF system as an important regulator of tumor stroma, we decided to examine the expression of PDGFR-*α* and PDGFR-*β* in both tumor and stromal cells in epithelial ovarian carcinomas and to investigate the possible relationship of the expression with histopathological characteristics and long-term overall survival.

## 2. Materials and Methods

### 2.1. Patients and Tissue Samples

Formalin-fixed, paraffin-embedded tumor specimens were obtained from a clinical study of patients with epithelial ovarian cancer, stages II to IV, who were enrolled in the Danish Ovarian Cancer Study Group (DACOVA) 9101 protocol from 1991 to 1994 [38]. The patients had undergone debulking surgery and were randomized to receive a combination of chemotherapy with either cyclophosphamide (500 mg/m^2^) and carboplatin at a dose of area under the curve 4 (AUC 4) in one arm or cyclophosphamide (500 mg/m^2^) and carboplatin at dose AUC 8 in the other arm. No survival difference between the two groups was observed. 

The paraffin-embedded formalin-fixed tissue and the slides from the primary operations were collected and underwent central review by a gynecopathologist. Details on this material have previously been published elsewhere [39]. The specimens were classified using the World Health Organization (WHO) histological classification 2003 and graded according to Shimizu et al. [40]. One-hundred and seventy cases were available for analysis.

### 2.2. Immunohistochemical Analyses

One representative tissue block containing tumor was selected from each patient and sections of 3-4 *μ*m were cut.The slides were immediately stored at −80°C until further use.

Rabbit polyclonal antibodies against PDGFR-*α* (Sc-338, dilution 1 : 200) and PDGFR-*β* (Sc-339, dilution 1 : 300, Santa Cruz Biotechnology, INC) were used as primary antibodies. The Dako Envision Flex Kit and Dako Rabbit Linker (K8002, K8005, and K8009, Dako, Glostrup, Denmark) were used for pretreatment and detection. Pretreatment for PDGFR-*α* was performed using the Target Retrieval Solution, high pH (pH 9), included in the Dako Envision Flex kit whereas pre-treatment for the PDGFR-*β* was performed in Target Retrieval Solution, low pH (pH 6.1), which is an additional reagent to the kit.

The Autostainer Plus Instrument (AS 10030; DAKO, Glostrup, Denmark) was used for the immunohistochemical staining starting with blocking of endogenous peroxidase, followed by incubation with primary antibody for 30 min, amplification with link antibody for 15 min, detection with HRP-conjugated polymer for 30 min, and finally visualization with DAB+. 

**Figure 1:**
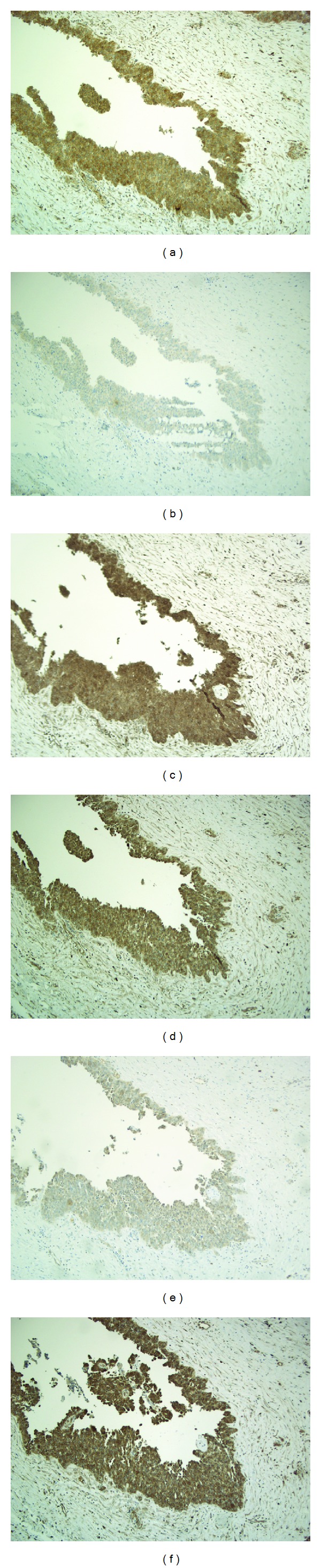
Examination of antibody specifities. Original magnification ×100. (a) Immunostaining with the primary antibody for PDGFR-*α* (SC-338) (b) Addition of blocking peptides for PDGFR-*α* (SC-338P) to the primary antibody for PDGFR-*α*, resulting in a significantly reduced staining reaction. (c) Addition of blocking peptide for PDGFR-*β* (SC-339P) to primary antibody for PDGFR-*α*, with no visible reduction in the staining intensity. (d) Immunostaining with the primary antibody for PDGFR-*β* (SC-339) (e) Addition of blocking peptides for PDGFR-*β* (SC-339P) to primary antibody for PDGFR-*β*, resulting in a significantly reduced staining reaction. (f) Addition of blocking peptide for PDGFR-*α* to antibody for PDGFR-*β*, with no visible reduction in the staining intensity.

The antibodies were tested with different pretreatment procedures and antibody dilutions to optimize the final staining protocol.The specifities of the antibodies were examined using blocking peptides for preadsorption for both PDGFR-*α* (SC-338P, Santa Cruz Biotechnology) and PDGFR-*β* (SC-339P, Santa Cruz Biotechnology), resulting in a significantly reduced staining reaction for each receptor as compared with the staining with the primary antibody. In order to compare the immunoreactivity from both receptors, blocking peptide for PDGFR-*α* was tested with the primary antibody for PDGFR-*β* and vice versa, and, as expected, there was no visible reduction in the staining intensity. This is demonstrated in Figure 1. Negative control slides without the primary antibody were run in every staining batch as well as positive tissue controls of the staining procedure consisting of tonsillar, appendix, and ovarian specimens. 

The immunohistochemical staining of tumor and stromal cells was evaluated separately and was scored by two of the authors (M. Waldstrøm and C. V. Madsen) independently and without knowledge of any of the clinicopathological data. In case of disagreement, the observers reexamined the slide together in order to establish a consensus score. Both intensity and percentage of positive cells were used for evaluating the immunoreactivity. In tumor epithelial cells, intensity was graded on a scale from 0 to 3. The extent of positively stained tumor cells was graded 0 for less than 1%, 0.1 for 1–9%, 0.5 for 10–50%, and 1 for more than 50% of the cells. A combined score was generated by multiplying the intensity and the extent. When the score value was above 1, the tumor was considered to have high expression. In the evaluation of the stromal cells, intensity was graded from 0 to 2, and extent was graded 0.5 for less than 50% and 1 for more than 50%. Expression was considered high when the score was above 1. 

Coexpression was defined when patients had high expression of both PDGFR-*α* and PDGFR-*β*.

### 2.3. Reproducibility of Immunohistochemical Scoring

The kappa values for interobserver agreement were moderate to substantial for the evaluation of PDGFR-*α* and PDGFR-*β* in tumor cells (kappa values between 0.60 and 0.64) and moderate for the evaluation in stromal cells (kappa between 0.48 and 0.51) [41]. One of the authors (C. V. Madsen) evaluated the slides twice with substantial intraobserver kappa values in tumor cells (kappa = 0.64–0.65) and moderate in stromal cells (kappa = 0.52–0.55).

### 2.4. Statistical Analyses

Kappa statistics were used for calculating the intra- and interobserver agreement of PDGFR expressions. Fisher's exact test or the chi-square test was used to examine the correlation among PDGFR expressions and clinicopathological parameters. Kaplan-Meier estimates were used for univariate overall survival analysis (OS), illustrated by survival plots, and logrank statistics were used for comparing the survival between the two groups. The initiating event was the time of diagnosis, and the endpoint of the overall survival analysis was death from any cause. *P* ≤ 0.05 was considered statistically significant. Statistical analyses were carried out using the NSCC software (Number Cruncher Statistical System, version 2007, Kaysville, Utah, USA).

## 3. Results

### 3.1. Patient Characteristics

Table 1 summarizes the patient characteristics. Serous adenocarcinoma was the most frequent histological subtype (78%), and high grade serous adenocarcinoma was seen in 63% of the patients. Most of the patients were diagnosed with FIGO III stage (74%). Median age of the women was 56 years. At the end of the follow-up period (December 2011), 21 patients remained alive. The median follow-up time for those patients was 19 years. 

Patients with serous adenocarcinoma grade II + III had a significantly lower overall survival than patients with serous adenocarcinoma grade I (*P* < 0.001).

### 3.2. Expression of PDGFR-*α* and PDGFR-*β* in Tumor and Stromal Cells

**Figure 2:**
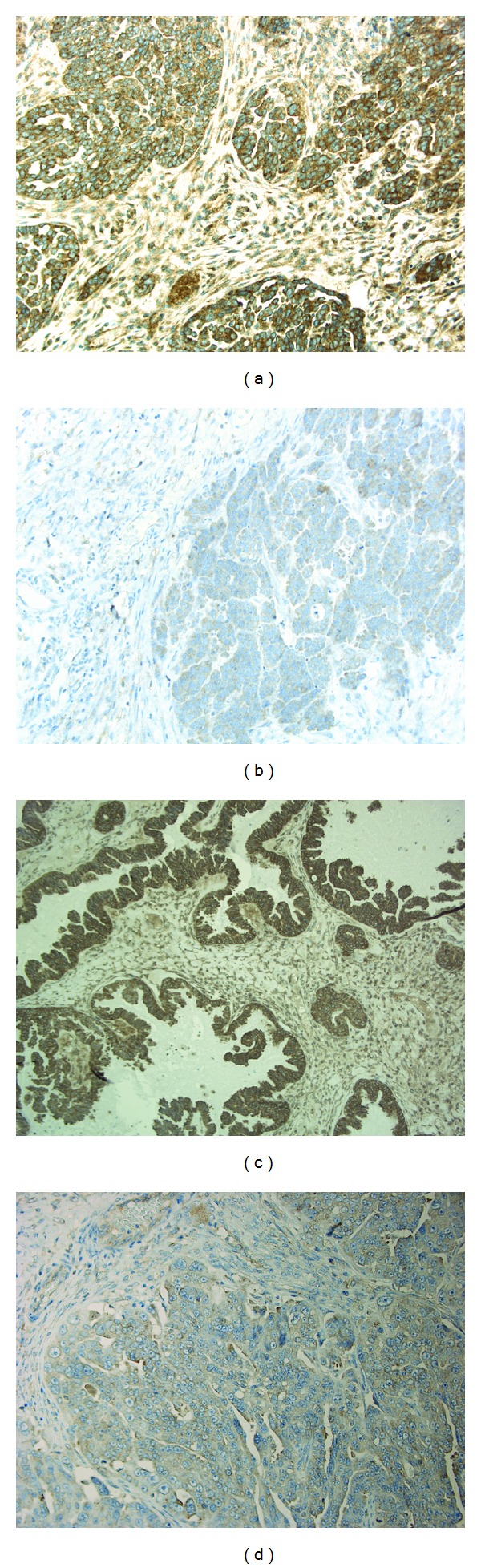
Immunohistochemical staining in ovarian carcinomas. Original magnification ×200. (a) High expression of PDGFR-*α* in tumor cells. (b) Low expression of PDGFR-*α*. (c) High expression of PDGFR-*β* in tumor cells. (d) Low expression of PDGFR-*β*. Example of high stromal reaction is seen in (a).

Figure 2 shows the immunohistochemical staining of PDGFR-*α* and PDGFR-*β* in tumor and stromal cells. The immunohistochemical staining of the receptors was cytoplasmic and membranous. 

High tumor cell expression of PDGFR-*α* or PDGFR-*β* was seen in 43% and 41% of the ovarian cancer specimens, respectively as summarized in Table 2. Coexpression of PDGFR-*α* and PDGFR-*β* was found in 37 (22%) of the samples (data not shown). 

Strong stromal reaction of PDGFR-*α* or PDGFR-*β* was seen in 32% and 44% of the ovarian cancer specimens, respectively, whereas co-expression of PDGFR-*α* and PDGFR-*β* was found in 33 (19%) of the ovarian cancer samples (data not shown). 

There was a significant association between patients with high expression of both PDGFR-*α* and PDGFR-*β* in tumor cells, *P* = 0.01. The same was seen between PDGFR-*α* and PDGFR-*β* in stromal cells, *P* = 0.003. Patients with high expression of PDGFR-*β* in stromal cells were more likely to have high expression of PDGFR-*β* in tumor cells, *P* < 0.001, and this was also seen for PDGFR-*α* in tumor and stromal cells, *P* < 0.001 (data not shown). 

### 3.3. Relation to Histopathological Characteristics and Clinical Outcome

Table 2 demonstrates the expression of PDGFR-*α* and PDGFR-*β* in tumor and stromal cells in relation to histological subtypes. The PDGFR-*α* and PDGFR-*β* expressions did not differ significantly between high grade (II + III) and low grade (I) serous adenocarcinoma in either stroma or tumor cells. Further statistical analysis of the receptor expression between mucinous, endometrioid, clear cell, and undifferentiated/mixed adenocarcinoma as regarding PDGFR expressions was not possible due to the low number of cases. However, it was noted that only few patients (≤33%) with mucinous adenocarcinoma (*n* = 12) had high expressions of PDGFR-*α* and PDGFR-*β* in both stromal and tumor cells.

Coexpression of both receptors in stromal cells was significantly higher in serous than in nonserous adenocarcinoma, *P* = 0.01, as shown in Table 3. There were no significant correlations between PDGFR-*α* and PDGFR-*β* expressions and grade, FIGO stage, and residual tumor. 

**Figure 3:**
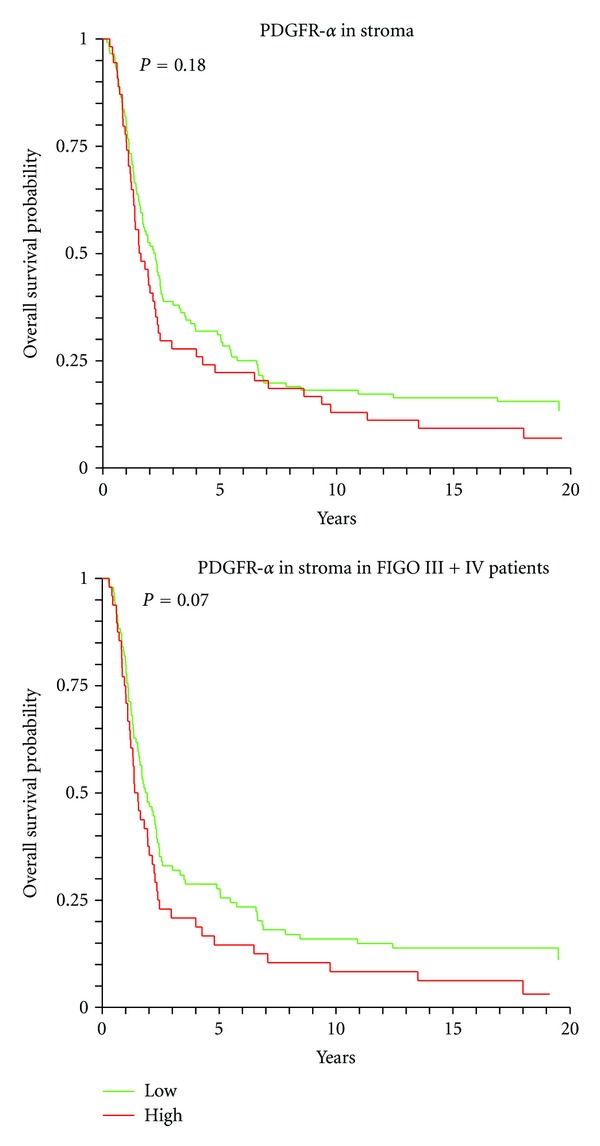
Overall survival curves for the expressions of PDGFR-*α* in stromal compartments.

There was a trend towards lower OS for patients with high stromal PDGFR-*α* expression, *P* = 0.18, as seen in Figure 3. This trend became stronger when focusing only on patients with FIGO III + IV, *P* = 0.07. There was no clear correlation between PDGFR-*α* expression and survival when focusing only on patients with high grade (II + III) serous adenocarcinoma. 

## 4. Discussion

Emerging evidence suggests that the PDGFsystem plays an essential role in carcinogenesis, also in ovarian cancer [37], and new biological agents targeting PDGFR are being investigated. The need for validated biomarkers that can be used in the stratification of patients for new treatment options is indeed urgent both from a patient perspective and from an economic point of view. Still, there are only a few published data on the PDGFR expressions in ovarian cancer. 

Our analyses demonstrated the presence of target for both PDGFR-*α* and PDGFR-*β* in the tumor and stroma compartments of a substantial proportion of the ovarian cancer samples. This is an important factor that needs to be taken into consideration when deciding on biological treatment targeting the PDGFsystem. The relation between high expression of the receptors in stroma and tumor cells may indicate that both systems are active in the same patients.

We used immunohistochemistry to evaluate the expression of the receptors which allows a semiquantitative evaluation of the expressions in stromal and tumor cells separately as well as in the subcellular compartments. The drawback of this technique is difficulties of standardization and reproducibility. The choice of antibody, staining procedure, scoring of the immunoreactivity, and different cut-off values to separate positive and negative reactions may account for some of the varying percentages of PDGFR expressions reported in ovarian cancer. Very few studies have reported kappa values during the investigation of PDGFR expression in ovarian cancer. Here, we demonstrated substantial kappa values of the PDGFR scoring in tumor cells, whereas the reproducibility was moderate with regard to the stromal cells. The explanation is in all probability that the staining reaction was more difficult to interpret.

A study by Köbel et al. [42] has clearly demonstrated that some biomarkers may be differently expressed in ovarian cancer depending on the histological subtypes of high-grade serous, low grade serous, endometrioid, mucinous, and clear cell, and their prognostic value may also be subtype-specific [42]. In the present study we did not find significant differences in expression of PDGFR-*α* and PDGFR-*β* between high grade (II + III) and low grade (I) serous adenocarcinoma but coexpression of PDGFR-*α* and PDGFR-*β* in stromal cells was seen more often in serous than in non-serous adenocarcinoma. 

One study has demonstrated PDGFR-*α* to be expressed more often in serous than in mucinous and endometrioid tumors [30], and another study has reported that none of the five mucinous adenocarcinomas included were positive for PDGFR-*α* or PDGFR-*β* [34]. This is in accordance with our findings where only a small number of the mucinous adenocarcinomas were positive for PDGFR-*α* or PDGFR-*β*. However, further studies are needed to clarify whether mucinous adenocarcinoma in general has low PDGFR expression. Two other studies have reported high expression of PDGFR-*α* and/or PDGFR-*β* in clear cell adenocarcinoma [32, 35]. We found high PDGFR-*β* expression in tumor cells in 60% of the clear cell adenocarcinomas, but no expression of PDGFR-*α*. However, in stromal cells 40% of the cases stained positive for PDGFR-*α*. As only five cases of this histological subtype were included in our study, it is difficult to draw conclusions. 

Previously, a relation between high PDGFR-*α* expression in the in tumor cells and lower survival has been reported in the studies by Henriksen et al. [30] and Lassus et al. [31]. We did not find the same relation although we noticed a tendency towards high expression of PDGFR-*α* in stromal cells and poor survival. The reason for these conflicting results could be the different methods used. It should be noted that Henriksen et al. performed the IHC analysis on fresh frozen specimens, whereas Lassus et al. performed the IHC analysis on tissue microarray and used only the intensity to assess the immunoreactivity. Regarding PDGFR-*β* and prognosis, a small study by Dabrow et al. suggested a relation between positive PDGFR-*β* expression in tumors and longer relapse free survival [29] but presented no data on overall survival, making a comparison with our results difficult.

In conclusion, the frequent expression of PDGF-receptors in ovarian carcinomas that has been found in the present study gives reason to believe, as suggested by previous studies, that the PDGF system plays a role in ovarian cancer. We found that co-expression of PDGFR-*α* and PDGFR-*β* in stromal cells was seen more often in serous adenocarcinomas than in non-serous adenocarcinomas. Although PDGFR-*α* or PDGFR-*β* did not show significant prognostic value as single markers in this material with long-term followup, the findings invite further studies exploring biological and clinical aspects of the PDGF system in ovarian cancer.

## Figures and Tables

**Table 1:** Patient characteristics.

Clinicopathological parameters	*N* = 170 (%)
Age	
25–59	112 (66)
60–89	58 (34)
FIGO stage	
II	28 (16)
III	125 (74)
IV	17 (10)
Histological tumor grade	
G1	40 (24)
G2	47 (28)
G3	68 (40)
Not graded	15 (9)
Histological cell type	
Serous	132 (78)
Grade I	26 (15)
Grade II + III	106 (63)
Mucinous	12 (7)
Endometrioid	11 (6)
Clear cell	5 (3)
Undiff/mixed	10 (6)
Residual tumor	
≤1 cm	67 (39)
≥1 cm	76 (45)
Unknown	27 (16)

**Table 2:** Expression of PDGFR-*α* and PDGFR-*β* and relation to histological characteristics *N* = 170.

	PDGFR-*α* low	PDGFR-*α* high		PDGFR-*β* low	PDGFR-*β* high
Tumor					
Total	97 (57)	73 (43)	170 (100)	101 (59)	69 (41)
Serous					
Grade I	13 (50)	13 (50)	26 (100)	11 (42)	15 (58)
Grade II + III	59 (56)	47 (44)	106 (100)	65 (61)	41 (39)
Mucinous	9 (75)	3 (25)	12 (100)	10 (83)	2 (17)
Endometrioid	4(36)	7 (64)	11 (100)	5 (46)	6 (54)
Clear cell	5 (100)	0 (0)	5 (100)	2 (40)	3(60)
Undiff/mixed	7 (70)	3 (30)	10 (100)	8 (80)	2 (20)

Stroma					
Total	116 (68)	54 (32)	170 (100)	95 (56)	75 (44)
Serous					
Grade I	18 (69)	8 (31)	26 (100)	13 (50)	13 (50)
Grade II + III	67 (63)	39 (37)	106 (100)	57 (54)	49 (46)
Mucinous	9 (75)	3 (25)	12 (100)	8 (67)	4 (33)
Endometrioid	9 (82)	2 (18)	11 (100)	6 (55)	5 (45)
Clear cell	3 (60)	2 (40)	5 (100)	5 (100)	0 (0)
Undiff/mixed	10 (100)	0 (0)	10 (100)	6 (60)	4 (40)

**Table 3:** Coexpression of PDGFR-*α* + PDGFR-*β* in relation to histological subtype.

Stromal	PDGFR-*α* + PDGFR-*β*		*P* value
	Low	High
Serous	101 (76.5)	31 (23.5)	0.01
Nonserous	36 (95)	2 (5)	
